# Monitoring air pollution close to a cement plant and in a multi-source industrial area through tree-ring analysis

**DOI:** 10.1007/s11356-021-14446-9

**Published:** 2021-05-27

**Authors:** Claudia Cocozza, Edoardo Alterio, Olivier Bachmann, Marcel Guillong, Tommaso Sitzia, Paolo Cherubini

**Affiliations:** 1 Department of Agriculture, Food, Environment and Forestry (DAGRI), Università degli Studi di Firenze, Via San Bonaventura 13, 50145 Florence, Italy; 2 Department of Land, Environment, Agriculture and Forestry, Università degli Studi di Padova, Viale dell’Università 16, 35020 Legnaro (PD), Italy; 3 Institute of Geochemistry and Petrology, ETH, Clausiusstrasse 25, 8092 Zurich, Switzerland; 4 WSL - Swiss Federal Institute for Forest, Snow and Landscape Research, Zürcherstrasse 111, 8903 Birmensdorf, Switzerland

**Keywords:** Tree rings, Environmental monitoring, Dendrochemistry, Laser ablation, Pollution forest archive

## Abstract

Thirty-two trace elements were examined in the tree rings of downy oak to evaluate the pollution levels close to a cement plant isolated in a rural context and an industrial area where multiple sources of air pollution are or were present. Tree cores were collected from trees growing 1 km from both the cement plant and the industrial area that are located 8 km from each other. The analysis of the trace elements was performed on annual tree rings from 1990 to 2016 using laser ablation inductively coupled plasma mass spectrometry. Trace elements Cs, Mg, Mn, S and Zn reflected the emission history of the cement plant. Their values have increased since early 2000s, when the cement plant started its activity. However, the lack of significant trends of pollutants in the tree rings from the industrial area and the possible effect of translocation and volatility of some elements left open questions. The very weak changes of the other trace elements in the period 1990–2016 suggest those elements do not mark any additional effect of the industrial activity on the background pollution. The results confirm that downy oak trees growing close to isolated industrial plants must be considered a pollution forest archive accessible through dendrochemistry.

**Supplementary Information:**

The online version contains supplementary material available at 10.1007/s11356-021-14446-9.

## Introduction

Industrial activities are important source of organic and inorganic pollution, including metals and metalloids that are released in the environment (Ali et al. 2019). The emissions from cement plants, for example, include heavy metals and organic compounds, such as polycyclic aromatic hydrocarbons as well as dust and other pollutants (Baldantoni et al. 2014; Rovira et al. 2014). Here, the emission of heavy metals is caused by the use of fuels, such as coal or solid wastes as supplementary substitute and many other processes associated with the production (Rovira et al. 2014). Although metals are blocked within the clinker, some of them are volatilized and condensed on the dust particles (Schuhmacher et al. 2009). Cadmium, Cr, Cu, Mn, Pb and Zn have been found in the emissions from cement plants (Schuhmacher et al. 2009; Bermudez et al. 2010; Ogunkunle and Fatoba 2014; Arfala et al. 2018). These pollutants can be transported by the wind and reach long-distance range, with important impact in the environment. Heavy metals are toxic pollutants altering ecosystem processes, accumulating in plants, animals and soils and thus negatively affect human health (Schuhmacher et al. 2009).

Trees continuously exposed to air pollution can uptake pollutants through the root, leaf and bark, allocating them in the wood (Lepp 1975). Since the 1970s, many studies have focused on the analysis of the chemical composition of tree rings: the so-called dendrochemistry (Ault et al. 1970; Symeonides 1979; Baes and Ragsdale 1981; Watmough and Hutchinson 1999). In recent years, many researches have been carried out, expecially in industrial areas, in order to study the chemical composition of trees and to find associations between industrial history and the pattern of chemical elements in tree rings (Martin et al. 2006; Aznar et al. 2008; Hojdová et al. 2011; Cui et al. 2013; Bernini et al. 2016; Cocozza et al. 2016; Odabasi et al. 2016; Sensuła et al. 2017; Perone et al. 2018; Liu et al. 2018; Austruy et al. 2019; Zhang 2019; Muñoz et al. 2019). Many of these studies have concerned the identification and the dating of the heavy metal pollution (Morton-Bermea et al. 2016). The usefulness of the temporal approach of dendrochemistry is that, in regions where monitoring stations suitable to monitor industrial plants are not present (or in places were they have limited records), trees can represent an alternative source of pollution data for the past decades, helping to trace the history of the chemical contamination (Alterio et al. 2020).

Many studies (Aznar et al. 2008; Hojdová et al. 2011; Sensuła et al. 2017; Liu et al. 2018; Perone et al. 2018) have demonstrated the feasibility of the dendrochemistry in the environmental monitoring and have shown the presence of similar patterns between the industrial history and the chemical content of tree rings. Howover, some others (Watmough and Hutchinson 2002; Martin et al. 2006; Navrátil et al. 2017) have highlighted the inconsistency of dendrochemistry in describing the chemical variation over time. Therefore, further dendrochemical studies (testing new and different geographical regions, tree species, chemical elements and industrial processes) are needed, in order to understand in detail the principles of dendrochemistry and to demonstrate its efficacy for tracing the chemical contamination in the environment (Liu et al. 2018). Moreover, these studies can provide valuable data for scientific reviews and meta-analysis, in order to identify gaps in knowledge about the reliability of dendrochemistry in environmental monitoring (Borenstein et al. 2011).

The Venafro plain in central Italy was considered as study case, focusing on a cement plant isolated in a rural context and 8 km away from a multi-source industrial area where different factories and sources of air pollution are placed. These factories had variable activities over time. In particular, a former foundry and an incinerator alternately worked from the 1970s until today, whereas the cement plant is active since the early 2000s. Since 2013, the Venafro plain has been defined as a highly polluted site in the Molise region (ARPA Molise 2020) because of road traffic in the town of Venafro and industrial pollution. The main objectives of this study are (a) to evaluate the cement plant and the industrial area as point sources of trace elements in tree rings and, consequently, (b) to assess if the trace elements in tree rings in each site refer to its emissions or not and if a higher number of pollution sources (as in the industrial area) can negatively affect on the reliability of the dendrochemical records.

## Materials and methods

### Study area

**Fig. 1:**
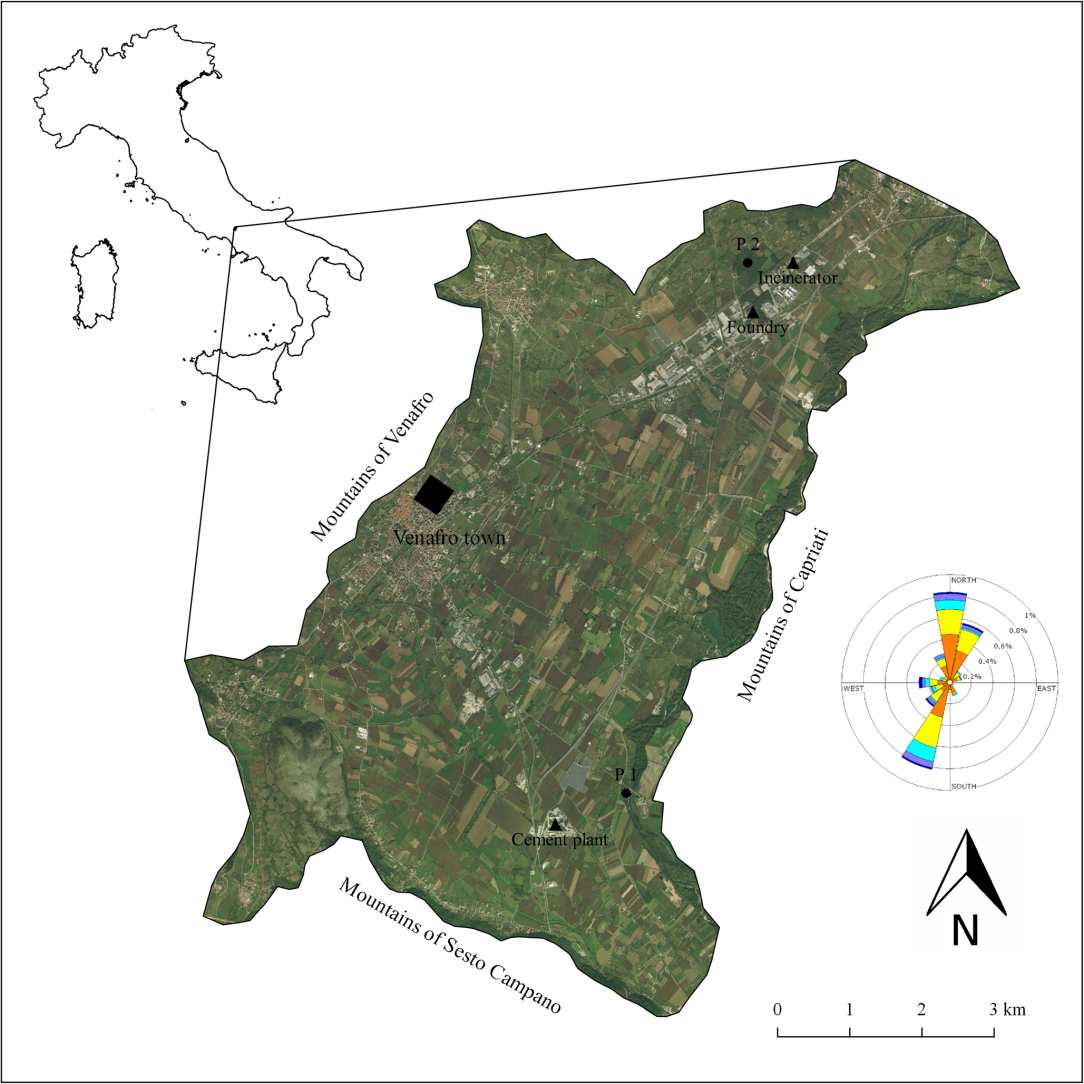
Study area, position of the cement plant, the foundry, the incinerator and the sampling sites P1 and P2. The wind rose shows the dominant winds direction in the study area

The study area is located in the Venafro plain, a small alluvial plain in central-southern Italy (Fig. 1). The most important human agglomeration in the area is the town of Venafro (41°28′57″ N, 14°02′51″ E; approximately 11,200 inhabitants). The plain covers an area of about 50 km^2^ and an altitude profile ranging between 130 and 250 m asl. The valley has a quasi-elliptical shape with the largest diameter arranged along the northeast-southwest axis and is surrounded by mountains (Lucenteforte 1877). The Venafro plain is a geological morphostructural depression, subsequently covered by river sediments of the Volturno river that flows on the east side of the valley (Amato et al. 2014). The main geological substrates are made up by alluvial deposits and compact calcareous rock layers (dolomites and travertines) (ISPRA 1983). The climate is temperate (14.5 °C mean annual air temperature, 1000–1200 mm mean annual precipitation) (Peel et al. 2007). The winds are predominantly from the NE or SW (ARPA Molise 2020) (Fig. 1).

The Venafro plain has around 30 factories that work in metallurgy, chemistry, cement production, electronics and waste incineration processes. Monitoring campaigns highlighted the low level of air quality in the valley. Indeed, the PMx levels in the Venafro town are very high, as recorded by the Regional Agency for the Environmental Protection (ARPA Molise 2020).

The study area is characterized by (i) a cement plant, located in rural context representing an isolated industrial plant, and (ii) an industrial area (8 km from the cement plant) that is characterized by multiple factories and sources of air pollution, including an incinerator and a foundry (Fig. 1).

The history of the industrial activities was temporally reconstructed through technical reports and other press sources.

**Fig. 2:**
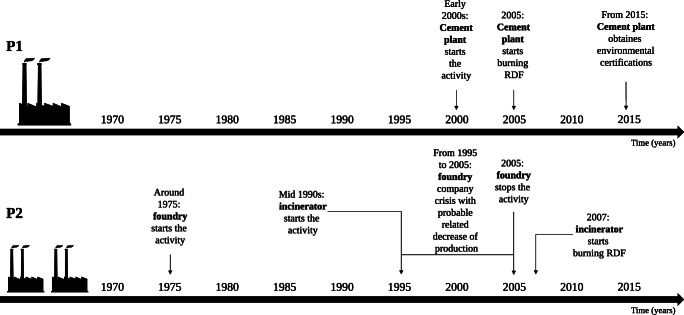
Industrial history in P1 and P2

1. The cement plant was established on early 2000s (2000–2001) (Sigas 2015). In 2005, the cement plant started to use refuse-derived fuel (RDF) in the combustion process (Colacem SpA 2011). In 2015 and 2016, this plant has obtained the Integrated Environmental Authorization (IEA) and the ISO 14001:2015 certification, respectively (Regione Molise 2015; Colacem SpA 2017).
2. The incinerator is still running since the mid of 1990s. Until 2005 it burned organic matter and biomass and since 2007 RDF (Herambiente SpA 2014). The plant was officially closed between 2005 and 2007.
3. The foundry has been active from the mid of 1970s to 2005, with periods of company crisis and probably related lower production from the mid of 1990s (La Banca 2014) (Fig. 2).

The cement plant and the incinerator are reported as sources of air pollutants in the European Pollutant Release and Transfer Register (E-PRTR) of European Environment Agency (2019) and the Italian Atlas of Environmental Conflicts (Atlante Italiano dei Conflitti Ambientali) by the Center for the Documentation on Environmental Conflicts (Centro Documentazione sui Conflitti Ambientali 2020). A biomonitoring program using lichens close to the incinerator detected the exceeding of heavy metals thresholds in 2011 and 2012 for As, Cu, Fe, Hg, Mn, Pb and V (ARPA Molise 2020).

### Tree-ring analysis

Trees of downy oak (*Quercus pubescens* Willd.) with average diameter at breast height (DBH) of 28 cm (± 6) and average age of 30 years (± 3) were selected in two sampling sites (P1 and P2). Three trees, two cores per tree, were collected in P1 and P2 sites. Oaks (and in particular the downy oak) are suitable indicators of the chemical contamination due to their low radial permeability, the small number of tree rings in the sapwood and the low heartwood moisture content (Cutter and Guyette 1993). P1 was located close to the cement plant and P2 close to the incinerator and the foundry placed in the industrial area (Fig. 1). Plots were at 1 km far from the emission sources (according to Cocozza et al. 2016; Perone et al. 2018).

Tree cores were collected at DBH using an incremental borer (Haglof Company Group, Sweden) in January 2018. In April 2018 tree cores were cut using a microtome (Gärtner and Nievergelt 2010) for dendrochemical analysis. In details, tree cores were not sanded, as usually done in dendrochronology, to avoid the contamination effect due to wood dust (Danek et al. 2015). The tree-ring width was measured using the LINTAB instrument (Rinntech, Heidelberg, Germany) and a Leica MS5 stereoscope (Leica Microsystems, Germany). The software TSAP Win 0.55 (Rinn 1996) was used to obtain raw tree-ring width chronologies and to statistically cross-date them in order to exactly identify the year of tree-ring formation (Speer 2012). Cross-dating was performed between the raw tree-ring width chronologies of each plot and between the mean chronologies of each plot (Perone et al. 2018). The *Gleichläufigkeit* statistical index and the relative significance value were calculated (Schweingruber 1988; Speer 2012). Moreover, the heartwood-sapwood boundary was identified. The delimitation was made through visual analysis based on the colour differences between heartwood and sapwood (the heartwood has a dark and distinctive colour while sapwood has a light colour) (Morais and Pereira 2012; Sohar et al. 2012). These analyses were conducted only on the tree cores used in the dendrochemical analysis (examples shown in Fig. S1).

### Dendrochemistry analysis

The analysis of chemical elements was performed on annual tree rings from 1990 to 2016 using the laser ablation inductively coupled plasma mass spectrometry (LA-ICP-MS) (Danek et al. 2015; Perone et al. 2018). Only one of the two cores collected from each tree was used in this analysis. The ablation was performed in spots, with one large spot (257 μm crater) in each tree ring. Wood samples were ablated orthogonally to the tree rings. Tree rings were ablated in the latewood where the presence of narrow-lumen and thick-walled cells defines conditions for a better combustion during the ablation (Danek et al. 2015). A summary of the main standard operating conditions and parameters used in the laser ablation analysis is reported in Table 1. Isotopes of elements, ^27^Al, ^137^Ba, ^209^Bi, ^79^Br, ^43^Ca, ^111^Cd, ^140^Ce, ^35^Cl, ^59^Co, ^53^Cr, ^133^Cs, ^63^Cu, ^65^Cu, ^57^Fe, ^39^K, ^25^Mg, ^55^Mn, ^95^Mo, ^23^Na, ^62^Ni, ^208^Pb, ^85^Rb, ^34^S, ^29^Si, ^88^Sr, ^232^Th, ^49^Ti, ^205^Tl, ^238^U, ^51^V, ^89^Y and ^66^Zn, were measured. The data were processed with the Sills software (Signal Integration for Laboratory Laser Systems) to select integration intervals, remove spikes and calculate net count rates (cps) for all measured elements (Guillong et al. 2008). Finally, the cps of each element were normalized to ^13^C to correct differences in ablation yield. Each analysis consisted of about 30 s of gas blank data, used for background correction and 40 s of sample ablation. Since the absolute concentrations were not calculated (because of the lack of a suitable reference material), the ratio between the cps of each element and ^13^C was taken as proxy for the element level in tree ring according to the formula:

$$ {l}_x^n=\frac{cps_x^n}{cps_x^{13_C}} $$

where ${l}_x^n$ is the level of the *n* element in the *x* year, ${cps}_x^n$ is the cps value of the *n* element in the *x* year and ${cps}_x^{13_C}$ is the cps value of ^13^C in the *x* year.

**Table 1:** Main information, standard operating conditions, and parameters of the laser ablation analysis

LA-ICP-MS analysis: parameters and description	
Instrument host	ETH Zurich
Type	Resolution 155S (asi)
ICP-MS	Element XR (Thermo Fisher)
Laser type	193 mm excimer
Setting	10 Hz
	3.5 J/cm^2^ single hole
	400 pulses
Spot size	257 μm of diameter
Normaliation	^13^C intensity

Subsequently, the level of elements was indexed (index level). In each tree core, the maximum and the minimum levels of each element were identified. Then, the time series of the index level were obtained according to the formula:

$$ {I}_x^n=\frac{\left({l}_x^n-{l}_{lowest}^n\right)}{\left({l}_{highest}^n-{l}_{lowest}^n\right)} $$

where ${I}_x^n$ is the index calculated in the *x* year for the *n* element, ${l}_x^n$ is the level of the *n* element in the *x* year and ${l}_{highest}^n$ and ${l}_{lowest}^n$ are the highest and the lowest level of the *n* element in the core, respectively (Perone et al. 2018). Time series of index level of tree cores of the same plot were averaged in order to obtain time series per plot (one in P1 and the other in P2).

### Statistical analysis

Time series of the index level were smoothed using a spline method (λ chosen with cross validation) (Wahba 1990; Aydin et al. 2013). The Kruskal-Wallis test was performed to test significant differences between the index levels of elements over time in relation to groups of years. In particular, the index levels were averaged in the following groups of years: 1990–1992, 1993–1995, 1996–1998, 1999–2001, 2002–2004, 2005–2007, 2008–2010, 2011–2013 and 2014–2016. Groups of years were tested as factors and were considered significantly different with *p* value ≤ 0.05, and, consequently, compared through LSD test.

The time series of index level were analysed to assess the trend of element level over time (increasing, decreasing, or no trend). The trend was estimated with the non-parametric Mann-Kendall test, obtaining the significance value (*p* value), the Kendall’s τ and the Kendall score (S) (Mann 1945; Hipel and McLeod 1994).

The S of a time series of values is:

$$ S=\sum \limits_{k=1}^{n-1}\sum \limits_{j=k+1}^n\mathit{\operatorname{sgn}}\left({x}_j-{x}_k\right) $$

where *x* are the time series values and where:

$$ \mathit{\operatorname{sgn}}(x)=\left\{\begin{array}{c}+1,\kern0.75em x>0\\ {}0,\kern0.75em x=0\\ {}-1,\kern0.75em x<0\end{array}\right. $$

An increasing trend is defined by positive values of S and a decreasing trend by negative values of S (Hipel and McLeod 1994). The Mann-Kendall test also provides the value of Kendall’s τ. Kendall’s τ is closely related to the S according to the formula:

$$ \tau =\frac{S}{D} $$

where *D* is the maximum value of *S* obtainable with the same series (*x*_*j*_ is always greater than *x*_*k*_). So, *τ* ranges from 1 (when *S = D* and the trend is always increasing) to −1 (when *S = D* but values are negative and the trend is always decreasing) passing through 0 (*S = 0*, no trend). This classification defines the correlation between series within time (Hipel and McLeod 1994). Statistical analyses were performed using SPSS statistical package (Version 20.0) (IBM corp. 2011) and R software (Version 3.5.2) (R Core Team 2018). Maps and graphics were produced using R software.

## Results

**Fig. 3:**
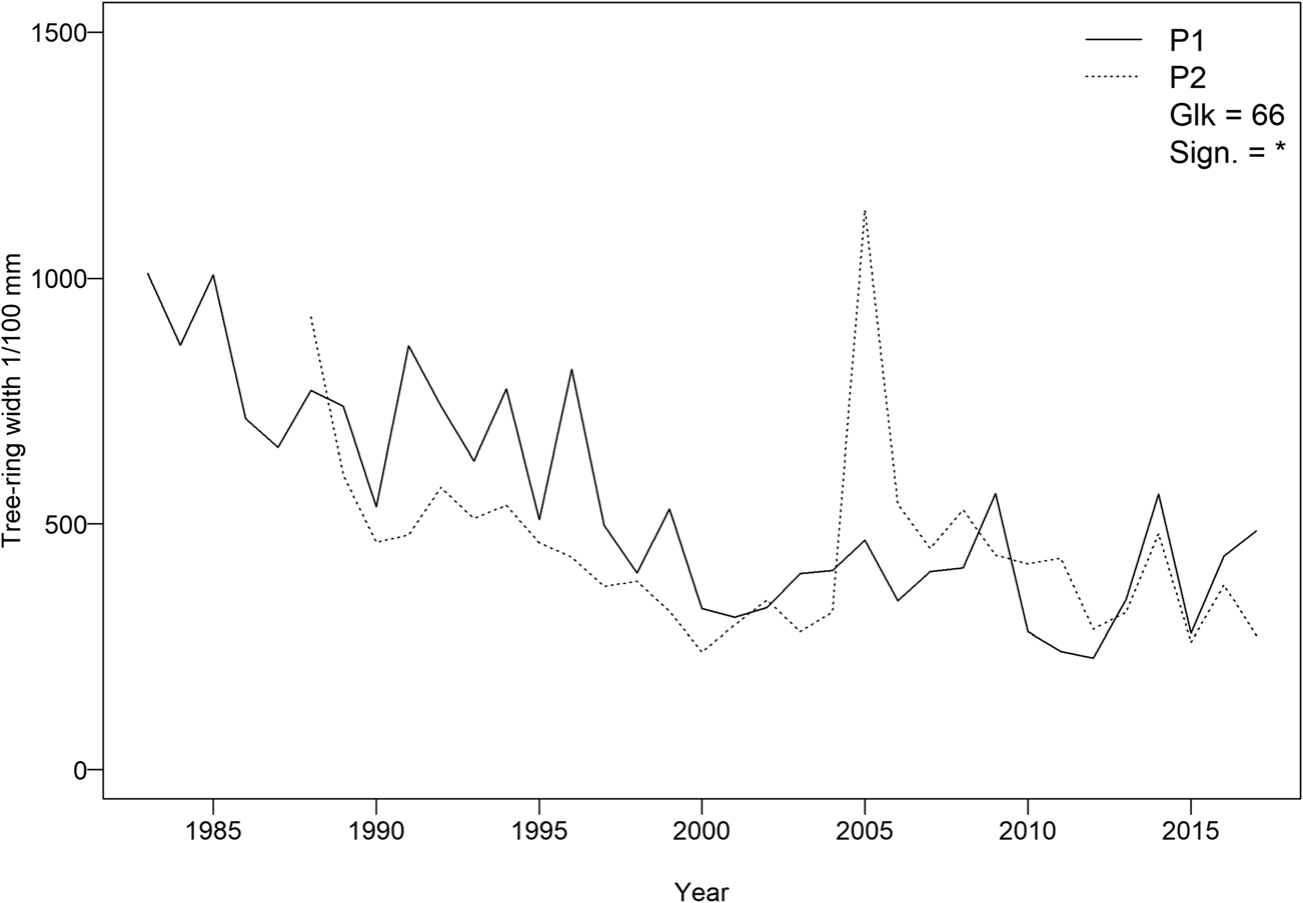
Mean tree-ring width chronologies of P1 and P2. Statistical values of the cross-dating (*Gleichläufigkeit* and significance) are given (significance level: * = 95%)

**Fig. 4:**
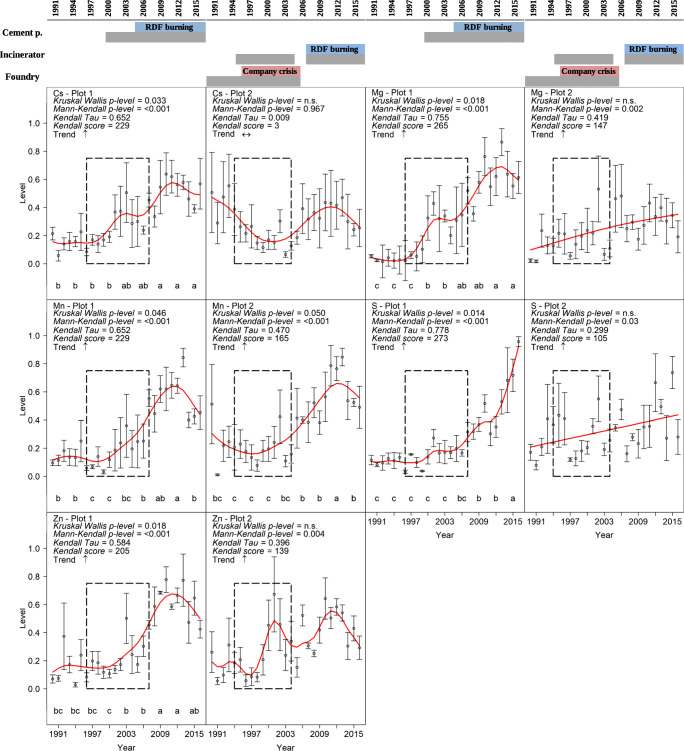
Trend over time of the level of Cs, Mg, Mn, S and Zn in P1 and P2 (values of Kruskal Wallis p-level, Mann-Kendall p-level, Kendall Tau, Kendall score and an arrow indicating the trend are reported). In graphs, circles are the mean (± standard error) chemical level of element per each year (*n* = 3), and letters (from LSD test) refer to the groups of 3 years considered in statistical analysis and red lines are smoothing functions. The black dashed boxes indicate the range (latest-oldest) of the years corresponding to the heartwood-sapwood transition zones recorded in each plot. In the upper part of the figure main information on the history of the industrial plants (in grey the activity years, see Fig. 2 for more details)

Mean tree-ring chronologies ranged from 1983 to 2017 in P1 (close to the cement plant) and from 1988 to 2017 in P2 (close to the industrial area). Mean tree-ring width was 4.97 mm (± 0.26 mm). Cross-dating between plot-mean chronologies was good, with a *Gleichläufigkeit* value of 66 (*p* < 0.05) (Fig. 3). The heartwood-sapwood boundary corresponded to a wide range of relative position among the sampled trees, with the limits associated to the tree rings from 1994 to 2008. In P1, the latest was found between 2007 and 2008, while the oldest was found between 1996 and 1997. In P2 the latest was between 2004 and 2005 and the oldest between 1994 and 1995 (Fig. 4 and Fig. S1).

Cesium, Mg, Mn, S and Zn showed significant trends in tree rings, in contrast to all the other elements (Al, Ba, Bi, Br, Ca, Cd, Ce, Cl, Co, Cr, Cu, Fe, K, Mo, Na, Ni, Pb, Rb, Si, Sr, Th, Ti, Tl, U, V and Y) (Fig. S2 and Fig. S3). According to the Kruskal-Wallis test, in P1 the index levels (thereafter levels) showed significant differences (*p* < 0.05) among the groups of years in Cs, Mg, Mn, S and Zn. (Fig. 4). The levels have been low from 1990 to 2000 and increased in more recent groups of years (recent time). In Cs, Mg, Mn and Zn, the peak level happened between 2011 and 2013. After this group of years, the level tended to decrease. On the other hand, S showed a continuous increasing trend that reached 0.699 in 2014–2016 from the low mean level of 0.052 in the years 1990–1992 (Fig. 4). In P2, the patterns of level in time were flat or with few weak peaks, resulting in shapes different from the ones observed in P1. Only Mn showed a significant difference of the level among the groups of years in the Kruskal-Wallis test (*p* < 0.05). According to the Mann-Kendall test, strong increasing trends were found in P1 in all the investigated elements. In P2, Mg, Mn, S and Zn showed increasing trend, whereas Cs showed no trends (Fig. 4).

## Discussion

The pollutants in the tree rings partially reflected the industrial activity in the study area. The cement plant emissions were recorded by the wood samples collected from the P1 sampling site, which were easily distinguishable from other pollution sources, namely, the emissions from the industrial area in P2. Moreover, almost all the elements analysed in this study are linked to the cement production. Magnesium is linked to the use of raw materials, as calcareous rocks (containing Mg), in the clinker production process (Schneider et al. 2011; Abdel Hameed et al. 2016). Sulphur is one of the main gases emitted by cement plants in form of SOx (Ruth 1998; Schuhmacher et al. 2004). Very high concentrations of Mn and Zn were found near cement plants by Schuhmacher et al. (2002) and by Al-Khashman and Shawabkeh (2006). Concerning the Cs (^133^Cs, stable isotope), the monitoring and detection in the environment are rarely performed due to its limited use in industry and manufacturing. The main cause of the presence of Cs in the air, water and soil is the erosion and weathering of rocks and minerals. However, Cs has also been detected in the fly ash of waste incinerators and coal burning power plants (Fernández et al. 1992; Mumma et al. 1990). Therefore, considering the use of both coals (coal or coke) and RDF for the energy production in the cement plant, Cs might be linked to the cement production. Cesium can be absorbed by trees, and it can be radially transported from the bark to stem wood through parenchyma, as showed by Aoki et al. (2017) in 3-year-old Japanese cedar seedlings. In P1, Cs, Mg, Mn, S and Zn showed very low levels until 2000. Since the early 2000s, the cement plant has operated around the P1 sampling site, and after 2000–2001, the levels of Cs, Mg, Mn, S and Zn in tree rings increased until 2011–2013. During 2014–2016 the levels of Cs, Mg, Mn and Zn have decreased, while S has showed a continuous increasing trend.

The overall pattern of chemical level in P1 seemed to be in accordance with the industrial history of the cement plant. Although at first only petroleum coke, coal and methane gas were used in combustion process of the cement plant, since 2005 the plant has activated the use of RDF in combustions (Colacem SpA 2011, 2017; Fig. 2). This change may have contributed to the increase of air pollution in the environment, as reflected by the level patterns in the tree rings. In addition to the potentially harmful combustibles mentioned above, spent tires have been also used as fuel in the cement plant (as reported in Caldiroli 2015). According to Carrasco et al. (2002), the use of spent tires in cement manufacturing cause an increase of emissions of S (SO_2_, + 24%) and heavy metals such as Zn (+ 487%) and Mn (+ 100%). In Cs, Mg, Mn and Zn, a maximum peak of level was found in 2013–2014, followed by a slight reduction in 2015–2016. This time pattern may match the implementation of some environmental certification programmes. Indeed, in 2015 and in 2016, the cement plant obtained the Integrated Environmental Authorization (IEA) and the ISO 14001:2015 certification, respectively (Regione Molise 2015; Colacem SpA 2017; Fig. 2). On the other hand, the continuous increasing trend shown by S, even after 2013–2014, leaves open questions, even if it matches with the statistics presented in recent technical reports published by the cement plant (Colacem SpA 2017).

The trends of trace elements in the P2 were different from P1. Only Mn pattern partially reflected the industrial history of the area. The foundry officially stopped its activity in 2005 after a period of manufacturing crisis since the mid of the 1990s (La Banca 2014) that probably caused lower production and, therefore, lower pollutant emissions. Then, in P2 the pollutant levels in tree rings were affected also by the incinerator. The incinerator intensified its activity in 2007 using RDF in combustion (Herambiente SpA 2014). Therefore, the period between 1995 and 2005 has been marked by the low emissions of foundry and not by incinerator, a change that could be traceable in the lower peaks of Mn in these years. Trace elements in tree rings from the P2 did not show significant trends related to the surrounding industrial history probably due to the wide diffusion of pollutants from the dense industrial area. Our results showed that the presence of several sources of pollution increases the noise in the tree-ring records.

Volatility of chemical species and heavy metals may affect their availability in the environment and, thus, their occurrence in tree wood. Volatility depends on the chemical element and the industrial/combustion process. Elements involved in volatilization process are those characterized by low boiling points. On the other hand, also semi-volatile elements may be strongly present in fly ashes even if a large portion generally remains in the bottom ashes (Abanades et al. 2015). In solid waste incinerator process, Zn is partially volatilized, while a small fraction can remain in the bottom ashes (Abanades et al. 2015). Pedersen et al. (2010) analysed the fate of several elements from the combustion of a dedicated waste and expected to contain high concentrations of potentially harmful elements such as heavy metals, S and Cl. They found that S and Zn were strongly volatilized. The release pattern depends on the combustion temperature: the higher is the temperature, the higher is the occurrence of these elements in the fly ashes. Conversely, Mg was not released, in accordance with its non-volatile nature. Another feature that may influence pollutant volatilization is the redox atmosphere during combustion. Indeed, it has been shown that a reductive combustion atmosphere favours the volatilization of Cd and Zn (Dong et al. 2015). Cesium is a semi-volatile toxic metal that can be released during the combustion of coal. It can vaporize in the hottest parts of the combustion elements (Seames and Wendt 2001). Manganese is a low volatile element. However, volatilization may happen, especially in coal combustion (Dıaz-Somoano and Martinez-Tarazona 2003; Tang et al. 2018). According to this framework, the five elements chosen to be discussed in this study show different volatility. Mg has non-volatile nature. Cs, Mn and Zn are semi-volatile elements, whereby the presence in the fly ashes, thus in the air, depends on several technical features during industrial process such as temperature and redox atmosphere during combustion. Conversely, S is a volatile element (Bøjer et al. 2008; Pedersen et al. 2010), converted into gaseous pollutants, especially during coal combustion (Hodges and Richards 1989; Folgueras et al. 2004; Schuhmacher et al. 2004; Buhre et al. 2006). In this work, S showed a strong increasing trend in P1, where coal and coke have been used as fuel in combustion process. However, the fate of the other elements leaves open questions about the consistency of chemical species with the background industrial history.

The element translocation throughout the xylem is a key issue in dendrochemistry (Alterio et al. 2020), because it may affect the reliability of dendrochemistry in environmental monitoring. Translocation means different xylem-xylem movement. Among them, the most common is the accumulation of elements in the transition zone, namely, the boundary layers between heartwood and sapwood (Cutter and Guyette 1993; Binda et al. 2021; Nováková et al. 2021). Considering the heartwood-sapwood transition zones recorded in each plot (as shown in Fig. 4 with black dashed boxes), the high peak showed by Zn in P2 and the weaker ones showed by Cs and Mg in P1 may be the result of an element accumulation into the heartwood-sapwood transition zone. Conversely, in the case of Cs and Mn in P2, the transition zone was characterized by the lowest values in the record. On the other hand, other translocation phenomena may have influenced the element trend, including translocation from heartwood to sapwood. The movement of elements could cause a reduction of the accuracy of the pollution time patterns, and thus it must be taken into account.

Nevertheless, the cement plant and the industrial area in the Venafro plain represent point sources of trace elements pollution, detectable in tree rings. In particular, the single pollution source (cement plant in P1) located in an agro-industrial land cover pattern (*sensu* Alterio et al. 2020), increased pollutants levels in tree rings consistently with the industrial history of the area, by showing considerably increased Cs, Mg, Mn, S and Zn levels in the recent years. On the other hand, the high variability of emitted pollutants in a multi-source industrial area did not define univocal pollutants levels in tree rings in P2, where the pollution sources are different and mixed, highlighting the difficulty to find unequivocal traces of the industrial history in the wood.

## Conclusions

Trace elements Cs, Mg, Mn, S and Zn had reflected the emission history of a cement plant isolated in a rural context. However, the lack of significant trends of pollutants in tree rings from an industrial area with multiple sources of air pollution and the possible effect of translocation and volatility of some elements left open questions by stimulating research in (a) the definition of the specificity of chemical species in the definition of pollutants in tree rings (does downy oak uptake all pollutants?) and (b) the assessment of threshold of pollutant concentration in the environment able to induce uptake in trees (what is the pollutant threshold that make the storage in tree rings detectable?). Moreover, further research should address the effect of pollutant source in tree rings, in order to define the pollutant level in the wood in relation to the distribution of trees and, consequently, to improve strategies in sampling design, as well as to assess the contribution of soil and groundwater in the pollutant accumulation in tree rings. Results from these researches would be incorporated into landscape or urban planning processes with the aim at protecting agricultural lands and humans living nearby and the trees that represent the elements of pollution forest archives.

## Supplementary Information

ESM 1 (DOCX 1537 kb)

## Data Availability

The datasets used and/or analysed during the current study are available from the corresponding author on reasonable request.

## References

[CR1] Abanades S, Flamant G, Gagnepain B, Gauthier D (2015). Fate of heavy metals during municipal solid waste incineration. Waste Manag Res.

[CR2] Abdel Hameed HM, Said A, Abdel Motalib A (2016). Applicability and characterization of clinker production from the raw materials of Sudr El-Hitan area, West Central Sinai, Egypt. Int J Innov Res Sci Eng.

[CR3] Ali H, Khan E, Ilahi I (2019). Environmental chemistry and ecotoxicology of hazardous heavy metals: Environmental persistence, toxicity, and bioaccumulation. J Chem.

[CR4] Al-Khashman OA, Shawabkeh RA (2006). Metals distribution in soils around the cement factory in southern Jordan. Environ Pollut.

[CR5] Alterio E, Cocozza C, Chirici G, Rizzi A, Sitzia T (2020). Preserving air pollution forest archives accessible through dendrochemistry. J Environ Manag.

[CR6] Amato V, Aucelli P, Cesarano M et al (2014) La valutazione delle deformazioni del suolo nella piana di Venafro mediante l ’ elaborazione di dati PSInSar , morfo-strutturali e stratigrafici. In: ASITA 2014. pp 51–53

[CR7] Aoki D, Asai R, Tomioka R, Matsushita Y, Asakura H, Tabuchi M, Fukushima K (2017). Translocation of 133Cs administered to Cryptomeria japonica wood. Sci Total Environ.

[CR8] Arfala Y, Douch J, Assabbane A, Kaaouachi K, Tian H, Hamdani M (2018). Assessment of heavy metals released into the air from the cement kilns co-burning waste: Case of Oujda cement manufacturing (Northeast Morocco). Sustain Environ Res.

[CR9] ARPA Molise (2020) Relazioni sulla qualità dell’aria. http://www.arpamoliseairquality.it/relazioni-sulla-qualita-dellaria/. Accessed 11 May 2020

[CR10] Ault WU, Senechal RG, Erlebach WE (1970). Isotopic composition as a natural tracer of lead in the environment. Environ Sci Technol.

[CR11] Austruy A, Yung L, Ambrosi JP, Girardclos O, Keller C, Angeletti B, Dron J, Chamaret P, Chalot M (2019). Evaluation of historical atmospheric pollution in an industrial area by dendrochemical approaches. Chemosphere.

[CR12] Aydin D, Memmedli M, Omay RE (2013). Smoothing parameter selection for nonparametric regression using smoothing spline. Eur J Pure Appl Math.

[CR13] Aznar JC, Richer-Laflèche M, Bégin C, Rodrigue R (2008). Spatiotemporal reconstruction of lead contamination using tree rings and organic soil layers. Sci Total Environ.

[CR14] Baes CF, Ragsdale HL (1981). Age-specific lead distribution in xylem rings of three tree genera in Atlanta, Georgia. Environ Pollut.

[CR15] Baldantoni D, De Nicola F, Alfani A (2014). Air biomonitoring of heavy metals and polycyclic aromatic hydrocarbons near a cement plant. Atmos Pollut Res.

[CR16] Bermudez GMA, Moreno M, Invernizzi R, Plá R, Pignata ML (2010). Heavy metal pollution in topsoils near a cement plant: The role of organic matter and distance to the source to predict total and HCl-extracted heavy metal concentrations. Chemosphere.

[CR17] Bernini R, Pelosi C, Carastro I, Venanzi R, di Filippo A, Piovesan G, Ronchi B, Danieli PP (2016). Dendrochemical investigation on hexachlorocyclohexane isomers (HCHs) in poplars by an integrated study of micro-Fourier transform infrared spectroscopy and gas chromatography. Trees - Struct Funct.

[CR18] Binda G, Di Iorio A, Monticelli D (2021). The what, how, why, and when of dendrochemistry: (paleo)environmental information from the chemical analysis of tree rings. Sci Total Environ.

[CR19] Bøjer M, Jensen PA, Frandsen F, Dam-Johansen K, Madsen OH, Lundtorp K (2008). Alkali/Chloride release during refuse incineration on a grate: Full-scale experimental findings. Fuel Process Technol.

[CR20] Borenstein M, Hedges LV, Higgins JPT, Rothstein HR (2011) Introduction to Meta-Analysis. Wiley

[CR21] Buhre BJP, Hinkley JT, Gupta RP (2006). Fine ash formation during combustion of pulverised coal-coal property impacts. Fuel.

[CR22] Caldiroli M (2015). Dal co-incenerimento dei rifiuti nei cementifici al “recupero energetico” con il combustibile solido secondario (CSS). Med Democr.

[CR23] Carrasco F, Bredin N, Heitz M (2002). Gaseous contaminant emissions as affected by burning scrap tires in cement manufacturing. J Environ Qual.

[CR24] Centro Documentazione sui Conflitti Ambientali (2020) Atlante Italiano dei Conflitti Ambientali. http://atlanteitaliano.cdca.it. Accessed 11 May 2020

[CR25] Cocozza C, Ravera S, Cherubini P, Lombardi F, Marchetti M, Tognetti R (2016). Integrated biomonitoring of airborne pollutants over space and time using tree rings, bark, leaves and epiphytic lichens. Urban For Urban Green.

[CR26] Colacem SpA (2011) Le Relazioni Costruiscono il Futuro-Rapporto di Sostenibilità 2011

[CR27] Colacem SpA (2017) Rapporto di sostenibilità 2017

[CR28] Cui M, He X, Davi N, Chen Z, Zhang X, Peng J, Chen W (2013). Evidence of century-scale environmental changes: Trace element in tree-ring from Fuling Mausoleum Shenyang, China. Dendrochronologia.

[CR29] Cutter BE, Guyette RP (1993). Anatomical, chemical, and ecological factors affecting tree species choice in dendrochemistry studies. J Environ Qual.

[CR30] Danek M, Bell T, Laroque CP (2015). Some considerations in the reconstruction of lead levels using laser ablation: Lessons from the design stage of dendrochemistry study, St.John’s, Canada. Geochronometria.

[CR31] Dıaz-Somoano M, Martinez-Tarazona MR (2003). Trace element evaporation during coal gasification based on a thermodynamic equilibrium calculation approach. Fuel.

[CR32] Dong J, Chi Y, Tang Y, Ni M, Nzihou A, Weiss-Hortala E, Huang Q (2015). Partitioning of heavy metals in municipal solid waste pyrolysis, gasification, and incineration. Energy Fuel.

[CR33] European Environment Agency (2019) European pollutant release and transfer register. https://prtr.eea.europa.eu/. Accessed 6 Jul 2019

[CR34] Fernández MA, Lluís M, Mercé S (1992). Behavior of heavy metals in the combustion gases of urban waste incinerators. Environ Sci Technol.

[CR35] Folgueras MB, Díaz RM, Xiberta J (2004). Sulphur retention during co-combustion of coal and sewage sludge. Fuel.

[CR36] Gärtner H, Nievergelt D (2010). The core-microtome: A new tool for surface preparation on cores and time series analysis of varying cell parameters. Dendrochronologia.

[CR37] Guillong M, Meier DL, Allan MM et al (2008) Sills: a Matlab-based program for the reduction of laser ablation ICP-MS data of homogeneous materials and inclusions. In: Mineralogical Association of Canada Short Course 40 40, Vancouver, B.C. pp 328–333

[CR38] Herambiente SpA (2014) Dichiarazione Ambientale-Sito di Pozzilli (IS)

[CR39] Hipel KW, McLeod AI (1994) Time series modelling of water resources and environmental systems. Elsevier

[CR40] Hodges NJ, Richards DG (1989). The fate of chlorine, sulphur, sodium, potassium, calcium and magnesium during the fluidized bed combustion of coal. Fuel.

[CR41] Hojdová M, Navrátil T, Rohovec J, Žák K, Vaněk A, Chrastný V, Bače R, Svoboda M (2011). Changes in mercury deposition in a mining and smelting region as recorded in tree rings. Water Air Soil Pollut.

[CR42] IBM corp. (2011) IBM SPSS Statistics for Windows

[CR43] ISPRA II for EP (1983) Carta geologica d’Italia alla scala 1:500.000

[CR44] La Banca ME (2014) Fonderghisa, la fabbrica dei veleni tra Pozzilli e Venafro. TerraMalata.it

[CR45] Lepp NW (1975). The potential of tree-ring analysis for monitoring heavy metal pollution patterns. Environ Pollut.

[CR46] Liu Y, Ta W, Cherubini P, Liu R, Wang Y, Sun C (2018). Elements content in tree rings from Xi’an, China and environmental variations in the past 30 years. Sci Total Environ.

[CR47] Lucenteforte FP (1877) Parte prima - stato fisico. In: Lucenteforte FP (ed) Monografia fisico-economico-morale di Venafro. Tipografia M. Cifarelli, Cassino, p 177

[CR48] Mann HB (1945). Nonparametric Tests Against Trend. Econometrica.

[CR49] Martin RR, Naftel SJ, Macfie SM, Jones KW, Feng H, Trembley C (2006). High variability of the metal content of tree growth rings as measured by synchrotron micro x-ray fluorescence spectrometry. X-Ray Spectrom.

[CR50] Morais MC, Pereira H (2012). Variation of extractives content in heartwood and sapwood of Eucalyptus globulus trees. Wood Sci Technol.

[CR51] Morton-Bermea O, Beramendi-Orosco L, Martínez-Reyes Á, Hernández-Álvarez E, González-Hernández G (2016). Increase in platinum group elements in Mexico City as revealed from growth rings of Taxodium mucronatum ten. Environ Geochem Health.

[CR52] Mumma RO, Raupach DC, Sahadewan K, Manos CG, Rutzket M, Kuntztt HT, Bache CA, Lisk DJ (1990). National survey of elements and radioactivity in municipal incinerator ashes. Arch Environ Contam Toxicol.

[CR53] Muñoz AA, Klock-Barría K, Sheppard PR, Aguilera-Betti I, Toledo-Guerrero I, Christie DA, Gorena T, Gallardo L, González-Reyes Á, Lara A, Lambert F, Gayo E, Barraza F, Chávez RO (2019). Multidecadal environmental pollution in a mega-industrial area in central Chile registered by tree rings. Sci Total Environ.

[CR54] Navrátil T, Šimeček M, Shanley JB, Rohovec J, Hojdová M, Houška J (2017). The history of mercury pollution near the Spolana chlor-alkali plant (Neratovice, Czech Republic) as recorded by Scots pine tree rings and other bioindicators. Sci Total Environ.

[CR55] Nováková T, Navrátil T, Demers JD, Roll M, Rohovec J (2021). Contrasting tree ring Hg records in two conifer species: Multi-site evidence of species-specific radial translocation effects in Scots pine versus European larch. Sci Total Environ.

[CR56] Odabasi M, Tolunay D, Kara M, Ozgunerge Falay E, Tuna G, Altiok H, Dumanoglu Y, Bayram A, Elbir T (2016). Investigation of spatial and historical variations of air pollution around an industrial region using trace and macro elements in tree components. Sci Total Environ.

[CR57] Ogunkunle CO, Fatoba PO (2014). Contamination and spatial distribution of heavy metals in topsoil surrounding a mega cement factory. Atmos Pollut Res.

[CR58] Pedersen AJ, Van Lith SC, Frandsen FJ (2010). Release to the gas phase of metals , S and Cl during combustion of dedicated waste fractions. Fuel Process Technol.

[CR59] Peel MC, Finlayson BL, McMahon TA (2007). Updated world map of the Koppen-Geiger climate classificatio. Hydrol Earth Syst Sci.

[CR60] Perone A, Cocozza C, Cherubini P, Bachmann O, Guillong M, Lasserre B, Marchetti M, Tognetti R (2018). Oak tree-rings record spatial-temporal pollution trends from different sources in Terni (Central Italy). Environ Pollut.

[CR61] R Core Team (2018) R: A language and environment for statistical computing. R Foundation for Statistical Computing

[CR62] Regione Molise (2015) Determinazione dirigenziale n. 16 del 21 luglio 2015

[CR63] Rinn F (1996) TSAP-Win. Time series analysis and presentation for dendrochronology and 409 related applications

[CR64] Rovira J, Nadal M, Schuhmacher M, Domingo JL (2014). Environmental levels of PCDD/Fs and metals around a cement plant in Catalonia, spain, before and after alternative fuel implementation. Assessment of human health risks. Sci Total Environ.

[CR65] Ruth LA (1998). Energy from municipal solid waste: A comparison with coal combustion technology. Prog Energy Combust Sci.

[CR66] Schneider M, Romer M, Tschudin M, Bolio H (2011). Sustainable cement production-present and future. Cem Concr Res.

[CR67] Schuhmacher M, Bocio A, Agramunt MC, Domingo JL, de Kok HAM (2002). PCDD/F and metal concentrations in soil and herbage samples collected in the vicinity of a cement plant. Chemosphere.

[CR68] Schuhmacher M, Domingo JL, Garreta J (2004). Pollutants emitted by a cement plant: Health risks for the population living in the neighborhood. Environ Res.

[CR69] Schuhmacher M, Nadal M, Domingo JL (2009). Environmental monitoring of PCDD/Fs and metals in the vicinity of a cement plant after using sewage sludge as a secondary fuel. Chemosphere.

[CR70] Schweingruber FH (1988) Tree rings: basics and applications of dendrochronology. Springer, Netherlands

[CR71] Seames WS, Wendt JOL (2001) The partitioning of arsenic, selenium, cadmium, and cesium during pulverized coal combustion in a 17 kW downflow combustor. Dev Chem Eng Miner Process 9:219–231. 10.1002/apj.5500090303

[CR72] Sensuła B, Wilczyński S, Monin L, Allan M, Pazdur A, Fagel N (2017). Variations of tree ring width and chemical composition of wood of pine growing in the area nearby chemical factories. Geochronometria.

[CR73] Sigas (2015) Report Sigas su cinque casi studio di conflitto ambientale in Italia

[CR74] Sohar K, Vitas A, Läänelaid A (2012). Dendrochronologia Sapwood estimates of pedunculate oak ( Quercus robur L .) in eastern Baltic. Dendrochronologia.

[CR75] Speer JH (2012) Fundamentals of Tree-Ring Research. University of Arizona Press

[CR76] Symeonides C (1979). Tree-ring analysis for tracing the history of pollution: application to a study in Northern Sweden. J Environ Qual.

[CR77] Tang Y, Guo X, Pan X, Finkelman R, Wang Y, Huan B, Wang S (2018). Changes and distribution of modes of occurrence of seventeen potentially-hazardous trace elements during entrained flow gasification of coals from Ningdong, China. Minerals.

[CR78] Wahba G (1990) Spline Models for Observational Data. Society for Industrial and Applied Mathematics

[CR79] Watmough SA, Hutchinson TC (1999). Change in the dendrochemistry of sacred fir close to Mexico City over the past 100 years. Environ Pollut.

[CR80] Watmough SA, Hutchinson TC (2002). Historical changes in lead concentrations in tree-rings of sycamore, oak and Scots pine in north-west England. Sci Total Environ.

[CR81] Zhang X (2019). The history of pollution elements in Zhengzhou , China recorded by tree rings. Dendrochronologia.

