# Supplementary material for: Monitoring air pollution close to a cement plant and in a multi-source industrial area through tree-ring analysis
Source: Environ Sci Pollut Res Int. 2021 May 27;28(38):54030–40. doi: 10.1007/s11356-021-14446-9 (PMC8476389; doi:10.1007/s11356-021-14446-9)
Supplement: Supplementary file 1 — (DOCX 1537 kb) [file 11356_2021_14446_MOESM1_ESM.docx]

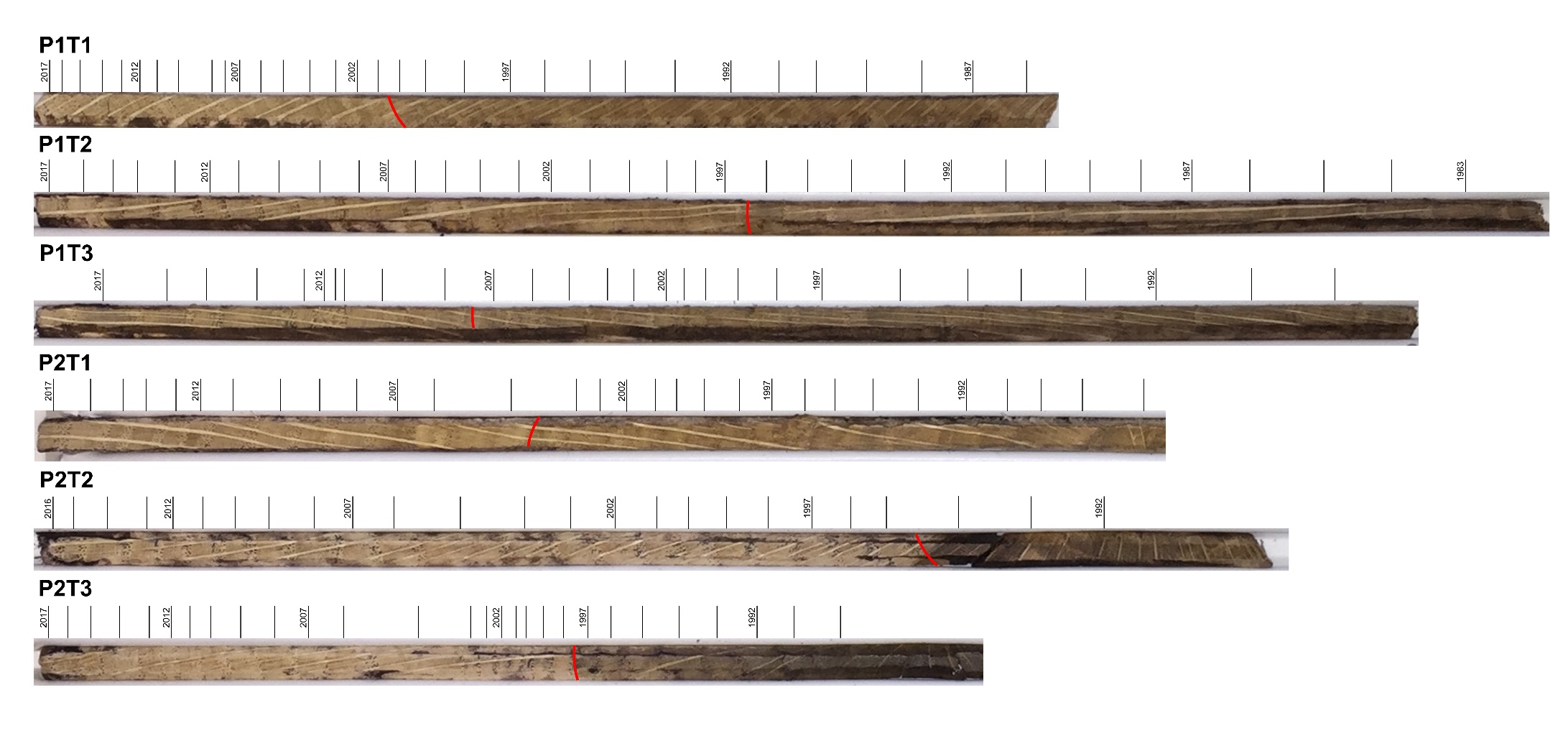


Figure S1. Photograhic images of the tree cores used in chemical analysis. The tree-rings years and the transition zone between heartwood and sapwood (red lines) are reported (P=plot; T=tree).


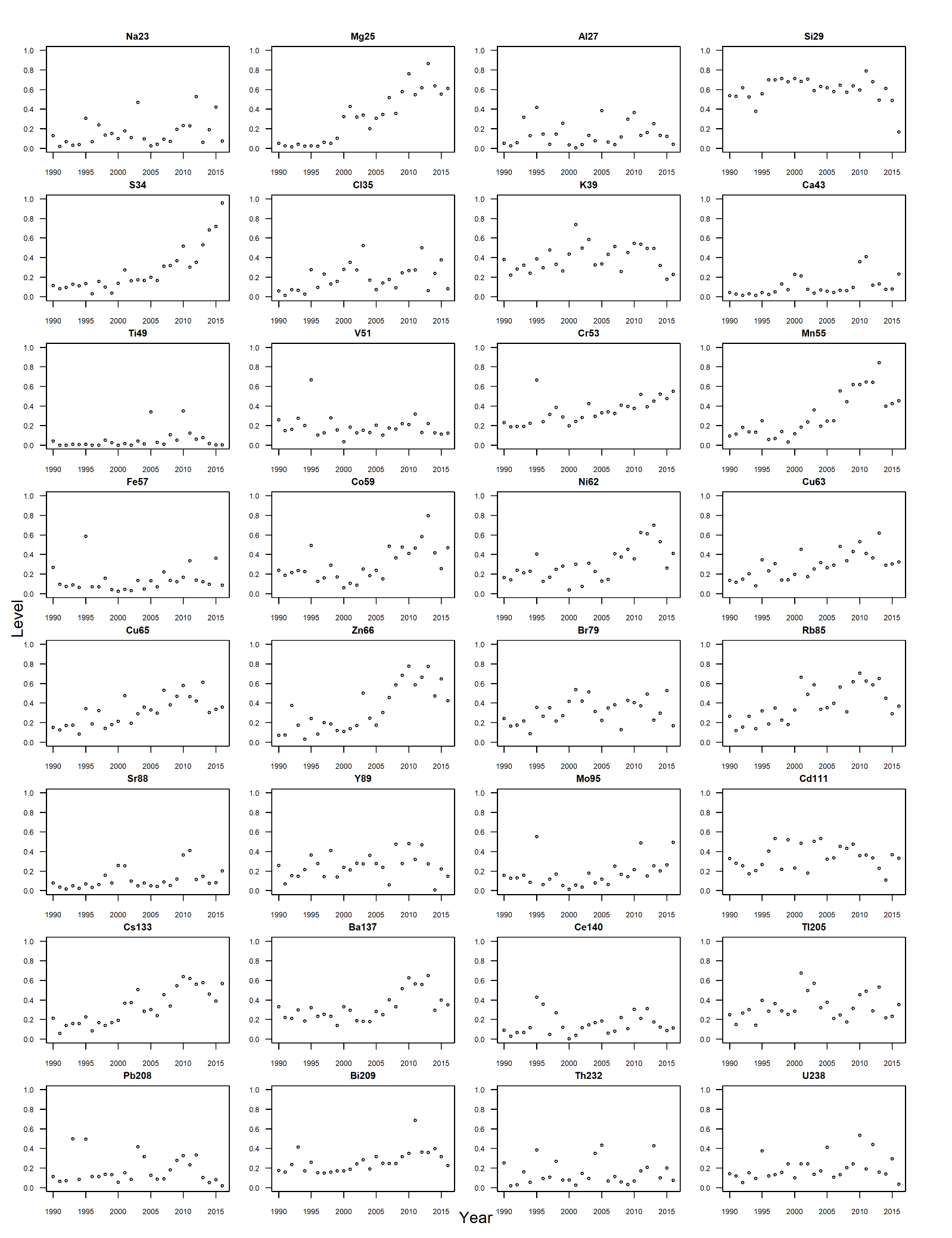


Figure S2. Trend over time of the level of the whole of the 32 elements investigated in this study in P1. circles are the mean chemical levels of element per each year. The main analysis of the study focus only on the 5 elements that showed statistical differences over time: Cs, Mg, Mn, S and Zn.


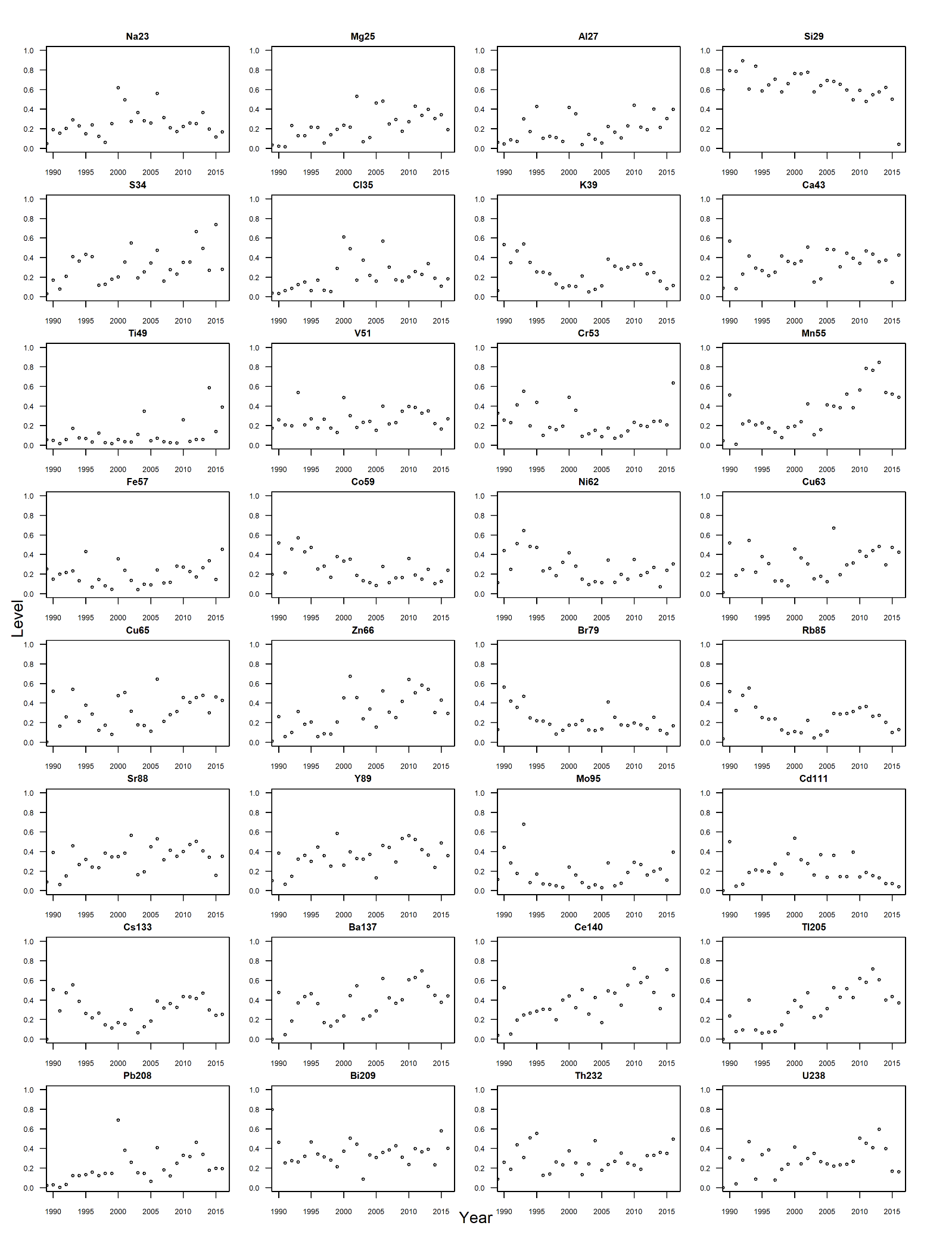


Figure S3. Trend over time of the level of the whole of the 32 elements investigated in this study in P2. circles are the mean chemical levels of element per each year. The main analysis of the study focus only on the 5 elements that showed statistical differences over time: Cs, Mg, Mn, S and Zn.
